# Modulation of erlotinib pharmacokinetics in mice by a novel cytochrome *P*450 3A4 inhibitor, BAS 100

**DOI:** 10.1038/sj.bjc.6604353

**Published:** 2008-05-06

**Authors:** N F Smith, S D Baker, F J Gonzalez, J W Harris, W D Figg, A Sparreboom

**Affiliations:** 1 Medical Oncology Branch, Center for Cancer Research, National Cancer Institute, 9000 Rockville Pike, Bethesda, MD 20892, USA; 2 The Sidney Kimmel Comprehensive Cancer Center at Johns Hopkins, 1650 Orleans Street, Baltimore, MD 21231, USA; 3 Laboratory of Metabolism, Centre for Cancer Research, National Cancer Institute, 9000 Rockville Pike, Bethesda, MD 20892, USA; 4 Bioavailability Systems, LLC, 2210 S Atlantic Avenue, Cocoa Beach, FL 32931, USA

**Keywords:** Tarceva, OSI-774, OSI-420, grapefruit, metabolism, *CYP3A4* transgenic

## Abstract

Administration of BAS 100, a novel mechanism-based CYP3A4 inhibitor isolated from grapefruit juice, resulted in a 2.1-fold increase in erlotinib exposure following oral administration to wild-type and humanised *CYP3A4* transgenic mice. This study illustrates the potential of BAS 100 to increase the low and variable oral bioavailability of erlotinib in cancer patients.

Erlotinib (also known as Tarceva or OSI-774) is a quinazolinamine, small molecule inhibitor of epidermal growth factor receptor (EGFR) tyrosine kinase. It is approved for the treatment of advanced nonsmall cell lung cancer as a single agent and advanced pancreatic cancer in combination with gemcitabine. Erlotinib has an average oral bioavailability in humans of 59% (Frohna *et al*, 2006), but exhibits substantial (up to eight-fold) interindividual pharmacokinetic variability (Hidalgo *et al*, 2001; Tan *et al*, 2004), which may result in variable treatment outcomes. Erlotinib is extensively metabolised into multiple products (Ling *et al*, 2006), including an active *O-*desmethyl metabolite, OSI-420. Cytochrome *P*450 3A4 (CYP3A4) plays a prominent role in the metabolism of this agent (Li *et al*, 2007b). As erlotinib is subject to extensive first-pass metabolism following oral administration, inhibition of intestinal and/or hepatic CYP3A4 activity may be a promising strategy to decrease interindividual pharmacokinetic variability. The objective of this study was to evaluate the effect of CYP3A4 inhibition by BAS 100, a novel spiro-ortho-ester mechanism-based inhibitor of CYP3A4 present in grapefruit juice (Li *et al*, 2006a, 2007a), on the pharmacokinetics of erlotinib in mice. Since the grapefruit effect was first reported in the early 1990s (Bailey *et al*, 1991), the ingestion of grapefruit juice has been shown to enhance the systemic exposure of a number of orally administered drugs (Bailey *et al*, 2004).

## MATERIALS AND METHODS

BAS 100 was obtained from Bioavailabilty Systems (Cocoa Beach, FL, USA), and erlotinib and OSI-420 were obtained from Toronto Research Chemicals (North York, ON, Canada). All other chemicals and reagents were purchased from Sigma-Aldrich (St Louis, MO, USA). Female BALB/c mice (6–8 weeks old) were kept in a controlled environment, with food and water available *ad libitum*. All procedures were carried out with NCI Animal Care and Use Committee approval. In the first study, BAS 100 and erlotinib were formulated in 10% DMSO and 5% polysorbate 80 in saline. Mice were randomised to receive erlotinib (10 mg kg^−1^, p.o.) alone or 30 min after BAS 100 (10 mg kg^−1^, p.o.). Blood was collected by cardiac puncture from three mice per time point at 0.083, 0.25, 0.5, 1, 2, 4, 6 and 24 h following erlotinib administration, and centrifuged to obtain plasma. In the second study, erlotinib was formulated in 0.3% carboxymethylcellulose and 0.1% polysorbate 80 in saline. Female *CYP3A4* transgenic mice (Granvil *et al*, 2003) and wild-type FVB/NCr mice (14 weeks old) received erlotinib (10 mg kg^−1^, p.o.) alone or 30 min after BAS 100 (25 mg kg^−1^, p.o.). Blood was collected 2 h after erlotinib administration. All samples were stored at −80°C until analysis. Plasma concentrations of erlotinib and OSI-420 were measured as described previously (Zhao *et al*, 2003). Plasma concentrations of BAS 100 were measured by a novel liquid chromatography-tandem mass spectrometry method. Briefly, plasma samples were prepared by protein precipitation with acetonitrile. BAS 100 and the internal standard (temazepam) were resolved isocratically on a Waters XTerra MS C18 column (50 × 2.1 mm internal diameter; 3.5 *μ*m particle size). The mass spectrometer was equipped with an electrospray ionisation source and operated in positive mode. Detection was performed by multiple reaction monitoring. The lower limit of quantitation was 10 ng ml^−1^. Pharmacokinetic parameters were determined by noncompartmental analysis using WinNonlin Professional Version 5.2 (Pharsight Corporation, Mountain View, CA, USA). Bailer's method (Bailer, 1988) was used to estimate the variance of the area under the curve (AUC). A *Z*-test was used for the pairwise comparison of AUCs (Yuan, 1993).

## RESULTS

**Figure 1:**
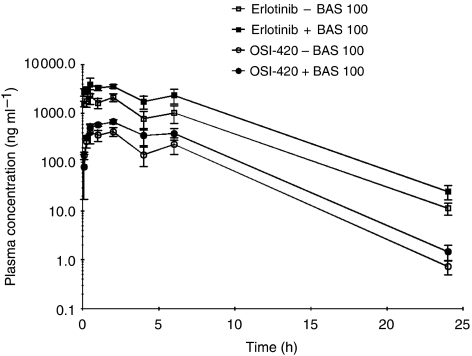
Plasma concentration–time curves of erlotinib and OSI-420 following administration of erlotinib (10 mg kg^−1^, p.o.) alone or 30 min after BAS 100 (10 mg kg^−1^, p.o.) to BALB/c mice. Data points and error bars represent the mean (*n*=3) and standard error, respectively.

**Figure 2:**
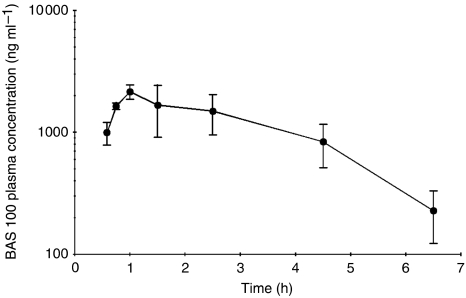
Plasma concentration–time curve of BAS 100. BAS 100 (10 mg kg^−1^, p.o) was administered to BALB/c mice 30 min prior to erlotinib (10 mg kg^−1^, p.o.). Data points and error bars represent the mean (*n*=3) and standard error, respectively.

Administration of BAS 100 prior to erlotinib resulted in a 2.1-fold increase in the AUC of erlotinib (37 953 *vs* 17 957 h ng ml^−1^, *P*<0.05, Figure 1). The AUC of the metabolite, OSI-420, was increased to a similar extent by BAS 100 (Table 1). The relative extent of metabolism of erlotinib into OSI-420 (0.21 and 0.17, respectively), and the half-life of OSI-420 (2.4 and 2.5 h, respectively) were similar whether erlotinib was administered alone or following BAS 100 (Table 1). Interestingly, a 2.5-fold increase in the dose of BAS 100 (to 25 mg kg^−1^) did not result in a further increase in erlotinib exposure (data not shown). The 2-h plasma concentration of erlotinib was increased by BAS 100 to a similar extent in *CYP3A4* transgenic (2.0-fold; 1857±300 *vs* 929±80 ng ml^−1^) and wild-type mice (1.9-fold; 1409±237 *vs* 744±135 ng ml^−1^). BAS 100 itself was orally bioavailable, reaching peak concentrations at 1 h following administration (Figure 2). These concentrations are in the range required for the inhibition of human CYP3A4 *in vitro* (Li *et al*, 2006a).

## DISCUSSION

The present study indicates that BAS 100 significantly increased the systemic exposure of erlotinib following oral administration, although it did not change the relative extent of metabolism of erlotinib into OSI-420. Furthermore, the half-lives of the parent drug and the metabolite were not affected by BAS 100. This suggests that the modulation of erlotinib pharmacokinetics by BAS 100 takes place at the level of intestinal absorption rather than elimination. The fact that BAS 100 increased circulating concentrations of erlotinib to a similar extent in mice expressing human *CYP3A4* in the intestine and wild-type mice further substantiates this supposition. However, it is also possible that, as a result of Cyp3a inhibition by BAS 100, biotransformation of erlotinib into OSI-420 is shunted to another metabolic pathway that is unaffected by BAS 100. In addition, it cannot be excluded that BAS 100 affects the formation of one or more of the other known erlotinib metabolites (Ling *et al*, 2006) that were not measured in this study, or that BAS 100 inhibits ABC transporter-mediated efflux of erlotinib in the intestine (Li *et al*, 2006a). Of note, the relative extent of metabolism of erlotinib into OSI-420 observed in this study is similar to that reported in cancer patients (0.12; Hidalgo *et al*, 2001). The bioavailability of erlotinib in mice has not been published. However, assuming it is similar to that observed in humans (59%; Frohna *et al*, 2006), one would not expect a modulator of its metabolism and/or transport to increase its exposure more than two-fold. Indeed, a 2.5-fold increase in the dose of BAS 100 (to 25 mg kg^−1^) did not result in a further increase in erlotinib exposure in the current study. In patients treated with erlotinib, the pharmacodynamic end point of rash, which has been associated with improved survival (Wacker *et al*, 2007), is significantly correlated to erlotinib exposure (Lu *et al*, 2006; Strother *et al*, 2006). Furthermore, previously obtained data on the related drug, gefitinib, indicate that patients with high CYP3A4 activity are likely to benefit from a modified regimen (increased dose), to achieve the drug concentrations required to interact with EGFR (Li *et al*, 2006b). A strategy by which enzyme activity is intentionally inhibited could achieve similar results. In view of the significant inverse correlation between decreasing absolute bioavailability and interindividual pharmacokinetic variability (Hellriegel *et al*, 1996), the current data provide a rationale for the development of exploratory clinical studies aimed at decreasing the variability in erlotinib exposure by concomitant administration of BAS 100.

## Figures and Tables

**Table 1:** Plasma pharmacokinetic parameters of erlotinib, OSI-420 and BAS 100 following administration of erlotinib (10 mg kg^−1^, p.o.) alone or 30 min after BAS 100 (10 mg kg^−1^, p.o.) to BALB/c mice

	***T***_**max**_ **(h)**	***C***_**max**_ **(ng ml^−1^)**	**AUC**_**last**_ **(h ng ml^−1^)**	**HL *λ***_**z**_ **(h)**
Erlotinib (−BAS 100)	0.5	2323	17957	3.1
Erlotinib (+BAS 100)	0.5	3952	37953	3.2
OSI-420 (−BAS 100)	2.0	430	3783	2.4
OSI-420 (+BAS 100)	2.0	682	6371	2.5
BAS 100 (+erlotinib)	1.0	2163	6940	1.8

AUC_last_=area under the curve from the time of dosing to the last measurable concentration; *C*_max_=maximum observed concentration; HL *λ*_z_=terminal half-life; OSI-420=*O-*desmethyl metabolite; *T*_max_=time of maximum observed concentration.
